# Mr. Barwell on the Tubercular Degeneration of the Products of Strumous Synovitis

**Published:** 1884-11

**Authors:** 


### Mr. Barwell on the Tubercular Degeneration of the Products of Strumous Synovitis.

After defining struma as "a condition usually inherited, which renders many of the tissues very prone to respond to slight sources of irritation by prolonged inflammations tending to suppuration and caseation," the author enters into the subject of the influence that this diathesis possesses over the course of certain forms of arthritis, particularly in reference to the degenerations of the new tissue, the product of the inflammation in strumous synovitis. "The new tissue has little or no tendency to cicatrization or fibrilliaton; it remains, or, at least, tends to remain for an indefinite time the same unformed or embryonic material, and then, without further organization, is apt to undergo either fatty or purulent degeneration. There is yet another degenerative process which must be noticed—the tuberculous." Remarking that for very many years the possibility or probability that the extreme obstinacy of strumous synovitis might be due to tuberculosis of the synovial membrane had been constantly borne in mind, the author refers to the investigations by Dr. Schüller on a number of animals which he had rendered diseased by the injection of tuberculous matter, and in which he had, at the same time, injured a knee-joint. In considering the effect that the results of these experiments should have on the theory of the tubercular origin of strumous synovitis, "that of a very slow malady commencing in children either a little weakened or depressed, or apparently in fairly good health, and who have received only a slight injury to the joint, or none at all," and the cases of the animals experimented on (generally rabbits prone to tubercular disease) "artificially affected with acute tubercular intoxication and at the same time with very severe traumatism of a joint," and that the latter were in a state by no means identical with that of a strumous child who has hereditary tendency to or is actually ailing from chronic tuberculosis, and who may have received slight or no articular injury, he examined the results of injections and contusions in twenty-four animals. Of these, five were injected with phthisical expectoration; these all showed changes considered to be tubercular; six were injected with minute pieces of tubercular lung; six with portions of tuberculous lymphatic gland; four were injected with lupus tissue; three with tuberculous synovial membrane. "Of the twenty-four injured and injected animals, we find that only those injected by sputum showed any clear marks of tubercle in the joint structures, save one dog injected with phthisical lung tissue." That the sputa injections should have had so much more effect on the injured joints than the injections of more tuberculous matter, he (Barwell) considered significant, and possibly due to the non-putrescence of that material.

"The experiments appear to show that, even while internal organs are deeply infected with acute and subacute artificial tuberculosis, the synovial membranes, even though severely injured, will resist the infection to such a degree that, as a rule, only 'doubtful' or 'initial signs' of tuberculous action can be detected by either naked eye or microscopic research. Moreover, it is a very instructive outcome of these experiments that in no single instance was a joint that had not been artificially injured affected with inflammation. * * * Does tubercle, then, ever become deposited in a previously healthy synovial membrane * and give rise to a typical inflammation? At present we have no evidence that this ever occurs, and Dr. Schüller's book goes very far to prove its non-occurrence, since

* Cancellous bone tissue stands in same conditions.

even inflamed synovial membranes were, with difficulty, infected, and since in the highly tubercularized animals which were the subjects of his experiments there was not found a single spot of tubercle in any one uninjured.

